# Cognitive Decline: Current Intervention Strategies and Integrative Therapeutic Approaches for Alzheimer’s Disease

**DOI:** 10.3390/brainsci14040298

**Published:** 2024-03-22

**Authors:** Kate S. Branigan, Blake T. Dotta

**Affiliations:** Behavioural Neuroscience & Biology Programs, School of Natural Science, Laurentian University, Sudbury, ON P3E2C6, Canada

**Keywords:** Alzheimer’s disease, electromagnetic fields (EMF), gamma oscillations, flickering light stimulation (FLS), cognitive functioning

## Abstract

Alzheimer’s disease (AD) represents a pressing global health challenge, with an anticipated surge in diagnoses over the next two decades. This progressive neurodegenerative disorder unfolds gradually, with observable symptoms emerging after two decades of imperceptible brain changes. While traditional therapeutic approaches, such as medication and cognitive therapy, remain standard in AD management, their limitations prompt exploration into novel integrative therapeutic approaches. Recent advancements in AD research focus on entraining gamma waves through innovative methods, such as light flickering and electromagnetic fields (EMF) stimulation. Flickering light stimulation (FLS) at 40 Hz has demonstrated significant reductions in AD pathologies in both mice and humans, providing improved cognitive functioning. Additionally, recent experiments have demonstrated that APOE mutations in mouse models substantially reduce tau pathologies, with microglial modulation playing a crucial role. EMFs have also been demonstrated to modulate microglia. The exploration of EMFs as a therapeutic approach is gaining significance, as many recent studies have showcased their potential to influence microglial responses. Th article concludes by speculating on the future directions of AD research, emphasizing the importance of ongoing efforts in understanding the complexities of AD pathogenesis through a holistic approach and developing interventions that hold promise for improved patient outcomes.

## 1. Introduction

Alzheimer’s disease (AD) is the most prevalent form of dementia and is a pervasive neurodegenerative disorder [1]. In 2010, there were approximately 35.6 million individuals diagnosed with dementia globally. This staggering number is predicted to double within the next two decades [2]. AD unfolds as a progressive degenerative ailment, with observable symptoms surfacing only after two decades of imperceptible changes in the brain [1]. These subtle changes culminate in noticeable deficits, prominently featuring memory loss and language problems. Such deficits stem from the deterioration and, in severe cases, the outright destruction of brain regions responsible for intricate processes like thinking, learning, and memory [1]. Once symptoms are apparent, the brain has run out of any compensatory mechanism to mask the disruption. The pathology of AD is marked by three significant abnormalities: brain atrophy, the formation of extracellular plaques through the aggregation of Amyloid-beta, and the accumulation of neurofibrillary tangles within still living yet affected neurons [3]. Despite decades of rigorous research, effective treatments or cures for AD remain elusive [1]. Beyond the clinical realm, the socio-economic impact of AD is substantial, affecting not only the individuals directly afflicted but also placing a considerable burden on healthcare systems and society as a whole. The urgency to understand the nature of AD pathogenesis and develop effective interventions is paramount.

Additionally, mild cognitive impairment (MCI) occupies a transitional position between typical age-related cognitive decline and AD [4]. Similar to AD, individuals with MCI may experience challenges in processing skills and cognitive functions such as learning, memory, language, and executive functioning [5]. However, the key distinction lies in the fact that individuals with MCI retain the ability to navigate daily life independently. Statistics suggest that approximately 20% of individuals aged 65 and above fall within the MCI category [6]. Recognizing this, early diagnostics play a crucial role in addressing MCI, as timely intervention proves to be the most effective approach in preventing the progression to severe cognitive impairment.

As researchers continue to research the complexities of MCI and AD, the pursuit of therapeutic breakthroughs remains a critical focus. This article delves into the multifaceted landscape of age-related cognitive decline, exploring the latest advancements in understanding its pathology and discussing emerging strategies in the search for viable treatments.

## 2. Cognitive Functioning and Brain Activity

Cognitive functioning encompasses mental processes essential for acquiring knowledge, manipulating information, and reasoning [7]. Each feature of cognitive functioning is associated with varying types of brain states and coherences [8]. The human brain functions through digital on-and-off signals known as action potentials, occurring in as little as 1 millisecond [9]. These events, when clustered together synchronously, are linked to thoughts, emotions, reconstruction of previous experience, and all cognitive processes [10]. We measure these action potentials using an electroencephalogram (EEG), which records brain waves in frequencies measured in Hertz (Hz). These frequencies range from very low (<4 Hz) during sleep to very high (>30 Hz) during higher order thinking and creativity [11]. These functional brain waves drive our behaviors. Cognitive functioning is influenced by brain waves, with high-frequency waves, known as gamma waves (>30 Hz), associated with higher brain functions like cognition and memory, and lower frequency waves, known as theta waves (4–8 Hz), linked to relaxation and low alertness [12,13]. These frequencies are of interest as gamma activity decline is noted in Alzheimer’s patients, affecting cognitive abilities [13]. 

Many other factors like neurological disorders, injuries, or psychological condition can produce alteration in cognitive functioning [14]. When these alterations occur quicker than anticipated, it can be considered MCI, where impairment in cognitive skills is greater than expected for an individual’s age but not severe enough to interfere significantly with daily life. Much like AD, MCI is associated with shifts in brain wave activity. A paper by Missonier and colleagues [15] demonstrated a disturbance in gamma-band dynamics in individuals with MCI. They found that those individuals who later experienced a decline in cognitive abilities displayed distinct brain wave patterns in the gamma band. These initial changes affected how the brain organized information during tasks that demand focused attention, implying a possible connection between these early deficits and the subsequent development of memory decline. Additionally, in the study by Goodman and colleagues [16] it was discovered that changes in theta–gamma wave coupling served as a predictor for memory performance in 99 participants. The results showed a performance hierarchy, with healthy volunteers surpassing those with MCI, and individuals with MCI outperforming those with AD. Moreover, the level of theta–gamma coupling followed the same order, and this coupling emerged as a significant predictor of memory scores.

The data demonstrates that healthy ageing produces anticipated changes in brain activity [8,17]. Individuals with MCI or AD have additional brain dynamic changes that can be best predicted by theta and gamma activity. We acknowledge that normal ageing, as well as MCI and AD, are associated with a host of cellular and protein differences as well. These changes can typically be masked or compensated by neighboring cells or biochemical pathways [18]. It is only in AD that this masking cannot occur. 

## 3. Traditional Therapies and Their Limitations 

There is currently no cure for AD, so the ongoing treatments are only symptomatic and have only modest benefits. The main priority has been to develop drugs with the potential to change the progression of the disease, rather than symptom management [19]. Currently there are only four drugs that are approved by the Food and Drug Administration (FDA) being utilized in the treatment of AD [19]. Three out of four of these drugs are inhibitors of the acetylcholinesterase enzyme (drugs: donepezil, galantamine, and rivastigmine). These drugs do not prevent neuronal loss, brain atrophy, and the progressive deterioration of cognition; they only contribute to small benefits in symptom management [19].

In more than fifty countries, donepezil is the leading medication for the treatment of AD. Donepezil is the number one member of the second generation of acetylcholinesterase inhibitors (AChEIs) which are inclusive of donepezil, rivastigmine, and galantamine [20]. The first generation of AChEIs included physostigmine, tacrine, velnacrine, and metrifonate, all of which led to pharmacokinetic and pharmacodynamic problems [20]. After which donepezil became the central component of AD therapeutics from 1996 up to now. Following 14 days after the administration of donepezil, the cerebral acetylcholine levels are increased by 35% and the acetylcholinesterase activity is decreased 66% and 32% in rat brain and blood, respectively. It is believed that the small and variable therapeutic effects of AChEIs are related to the pharmacological properties and individual capacity to inhibit AChE activity in the brains of patients with AD [20]. The majority of clinical trials involving donepezil in AD during the past 10 years have only been performed in patients with mild-to-moderate dementia and a very small number of studies have been with severe cases of AD. It has been concluded that donepezil is beneficial in a dose-dependent manner, but it has been determined that neither donepezil, rivastigmine, and galantamine are greatly efficacious [20].

AChEIs are being used because it has been hypothesized that the progressive loss of limbic and neocortical cholinergic innervation in AD is critically important for memory, learning, attention, and the decline of other higher brain functions [21]. It is believed that neurofibrillary degeneration in the basal forebrain is the primary cause for the dysfunction and death of cholinergic neurons in this specific region. This gives rise to widespread presynaptic cholinergic denervation [21]. Using AChEIs increases the availability of acetylcholine at synapses and has been clinically proven to be useful in delaying cognitive decline in AD [21].

However, AD remains incurable. The is due to the fact that when significant cognitive loss occurs, the neuronal networks that control the perturbed cognitive abilities are either dead or irreversibly damaged. Even if it were possible, replacing these dead and/or damaged neuronal networks would not reconstruct the intellectual identity of the patient affected with the disease [22]. There are currently neurocognitive and neuroimaging tests that are used with partial success in identifying people at a higher risk of AD; however, these tests cannot pinpoint a cause or specific intervention that could attenuate the progress of the disease [22].

The N-methyl-D-aspartate receptor (NMDAR) is pivotal in synaptic transmission and plasticity, believed to be fundamental to learning and memory [23,24]. The NMDAR is central to both the function and development of the central nervous system, as well as neurotoxicity [23]. Recently, NMDAR activation has been connected to AD-related synaptic dysfunction. Excessive calcium signaling leads to a gradual deterioration in synaptic function and eventual death of neurons. This aligns with the cognitive and memory decline seen in AD patients, as well as the development of abnormal neural structures [23,24]. For these reasons, memantine, which is an NMDAR antagonist, has been used in clinical trials as a symptomatic and neuroprotective treatment for AD [23,24]. Memantine is approved for moderate to severe cases of AD and is commonly prescribed to alleviate symptoms and enhance quality of life. Furthermore, memantine has shown limited success in halting or preventing hippocampal or total brain atrophy [23,24]. The advancement of AD can also be slowed via the use of cognitive enhancers, but only for a period of six months on average [25]. Although these medications can prove beneficial for small periods, eventually they need to be discontinued due to the adverse effects.

For over 20 years, immunization against beta-amyloid has been explored as a potential vaccination strategy to combat AD [26,27]. The current evidence supports multiple hypotheses that the accumulation and exceeding a threshold of amyloid-beta deposition in the brain is responsible for driving disease progression [26]. No vaccine has been licensed thus far and the therapeutic route of immunotherapy has come under considerable criticism, which is attributable to negative results yielded from several clinical trials [26,27].

With the attempt to use immunotherapy against AD, scientists have attempted to vaccinate against the beta-amyloid peptide, due to its central role in the pathogenesis of the disease [27]. Efforts have been made to reduce levels of beta-amyloid monomers, oligomers, aggregates, and plaques through the use of compounds aimed at decreasing production, inhibiting aggregation, or enhancing brain clearance of beta-amyloid [27].This course of action relies on the concept that the antibodies working against beta-amyloid have the capacity to interfere with its aggregation and accumulation, block its toxicity, or increase its catabolism. This is also reliant on the fact that these effects on the brain would modify the course of the disease [27].

Another confounding and important issue to this potential line of treatment is that because this is a vaccine for a non-infectious disease, herd immunity does not apply and the interindividual variability in both the magnitude and quality of the immune response plays a large role in whether this treatment is successful or not [27]. There is still no licensed anti-beta-amyloid vaccine or monoclonal antibody for AD, the immunotherapy against amyloid-beta in clinical trials has repeatedly been unsuccessful [26,27].

A final thread of current research focuses on biomaterials and bioengineering. Current therapeutic trials are replacing neurons that have been lost in the neurodegenerative phases of AD with stem cells. Stem cell-based therapy is both a suitable and effective restorative strategy for AD treatment as well as several neurodegenerative diseases, based on the neurogenesis capacities that stem cells hold [28,29]. There is a significant role played by intercellular binding proteins and glial cells when setting the extracellular conditions of neurons. The extensive deterioration of astrocytes, oligodendrocytes, and microglia, which all provide strength to the neuronal networks in the central nervous system, contributes to the extensive neuronal loss associated with AD [28,29]. The regeneration or transplantation, in situ, of destroyed neurons has provided anticipation of the restructuring of the central nervous system’s reliability and reducing the deterioration of the mental ability of patients with AD [28,29]. Bioengineering and biomaterials research in AD holds immense promise in revolutionizing therapeutic interventions and understanding the underlying mechanisms of the disease. Biomaterials play a crucial role in the design and fabrication of novel drug delivery systems, including nanoparticles and hydrogels, enabling targeted delivery of therapeutic agents to the brain while minimizing systemic side effects [28,29]. Furthermore, bioengineered models, such as organoids and microfluidic devices, offer powerful platforms for studying disease pathology and screening potential drug candidates in a controlled and physiologically relevant environment. Integration of cutting-edge technologies, such as artificial intelligence and microfluidics, with biomaterials holds the potential to accelerate drug discovery and facilitate the development of precision medicine approaches tailored to individual patients. The multitude of therapeutic and research interventions outlined above outline the necessity of combination therapy in effectively treating AD. Given the complex nature of the disease and the various factors influencing its progression, a singular treatment approach is often insufficient. Despite a notable increase in Alzheimer’s-related deaths over the past two decades, a definitive treatment plan remains elusive [28,29]. Thus, the imperative for combination therapy arises from the recognition that addressing multiple aspects of AD pathology and symptomatology simultaneously is crucial for achieving meaningful therapeutic outcomes. The FDA-approved therapies currently being utilized have not shown any remedial effect and have yet reduced the pace of the disease progression. Due to the multi-complexity of Alzheimer’s pathology, it impedes the advancement of therapeutic strategies. Several drugs and treatment strategies have been proven unsuccessful in regard to improving cognitive in minor-to-intermediate AD patients because the drugs only focus on a specific pathology, without recognizing other neuropathological conditions that define AD [28,29].

## 4. Alzheimer’s Disease Diagnostics

AD is typically diagnosed when patients acquired cognitive impairments have become severe enough to compromise social and/or occupational functioning. In contrast, MCI on the other hand, can be easily diagnosed with brief tests such as the Short Test of Mental Status, the Montreal Cognitive Assessment (MoCA), or the Mini-Mental State Examination (MMSE) [30]. The diagnostic accuracy for AD is much lower at earlier and presymptomatic stages, due to the nature of AD diagnosis being imprecise. A diagnosis of AD can only be definite with an autopsy [31]. The nature of intervention with AD is more efficacious before significant neurodegeneration has occurred, there is a great need for biomarker-based tests which will provide a more accurate and early diagnosis of AD. This would allow for improved monitoring of the disease progression [31]. The trademark defining lesions of AD are neurofibrillary tangles, made up of tau protein, and senile plaques that are formed from deposits of amyloid-beta. As a result, cerebrospinal fluid (CSF), tau protein, and amyloid-beta have been identified as being the most promising and informative biomarkers for AD [31]. If there are elevated levels of tau protein in CSF this is thought to occur after its release from both damaged and dying neurons which were connected to dystrophic tau neurites and tangles [31]. Decreased levels of amyloid-beta in CSF are thought to be a result of a large-scale accumulation of the least soluble amyloid-beta peptides into insoluble plaques in the brain with AD. Recent studies have postulated that the combination of elevated tau protein and decreased amyloid-beta in the CSF is a pathological biomarker signature that can be used as a diagnostic for AD [31].

Biomarkers are an essential part of disease treatments as they are essential for diagnosis, ability to monitor the disease progression, detecting early onset of the disease, and monitoring the effect of therapeutic intervention, as well as avoiding false diagnosis of the disease [32]. The characteristics of an ideal biomarker is that they should be highly specific, have the ability to predict the course of the disease accurately, and should reflect the degree of response to treatment of the disease [32]. The reason why CSF is considered a better source for biomarkers in AD is because it has direct contact with the extracellular space of the brain as well as having the ability to reflect biochemical changes that occur inside the brain. Unfortunately, none of the biomarkers for AD that are currently available are able to single-handedly accomplish the disease diagnosis [32]. Measuring biomarkers via a patient’s CSF does have some limitations, as it is extremely invasive. The sample of CSF has to be collected through lumbar puncture. Due to sample storage and transportation, there is an irreproducible diagnosis [32].

Neuroimaging tools are a technique that immediately provide structural and functional details of the brain, while also being helpful to predict and monitor disease progression of the consequences of AD neurodegeneration is the loss of brain volume, which can be differentiated from a normal brain by using computerized tomography (CT) and magnetic resonance imaging (MRI) techniques [32]. 

CT scans utilize radiation to produce images of both the brain and body. A head CT will show the shrinkage of certain brain structures, occurring as a consequence of dementia, specifically AD [33]. The reason that structural magnetic resonance imaging (sMRI) has been utilized to investigate AD is because it has the capacity to characterize brain volumes, areas, cortical thickness, and curvature, which are all useful measurements to monitor the presence and progression of the disease [34].

Using the CT and MRI techniques, it has been possible to show neuronal loss, the atrophy of medial temporal regions, and even neurofibrillary tangles in the brains of patients with AD [32]. It is now possible to distinguish atrophy during the early stage of AD compared to the atrophy of normal aging, using the MRI technique. MRI has the ability to reveal disease progression from cognitive normalcy to mild cognitive impairment to AD [32].

Cognitive processing requires the temporal coordination of widely distributed neuronal populations in the brain, these interactions utilize transmembrane currents, which is reflected in voltage fluctuations which can be measured at multiple different levels, one being cortical via the use of an EEG [35]. EEGs measure electrical activity non-invasively by means of electrodes that are placed on the scalp. Recent studies have utilized EEGs as means of potential diagnosis and disease monitoring of AD by studying three categories using EEGs: change in frequency pattern, reduction in the complexity of the EEG signals, and perturbation in EEG synchrony [35]. While using an EEG it is important to distinguish changes that may appear in mild cognitive impairments and AD from those that appear due to normal aging. Studies have confirmed that the alpha rhythm in posterior cortical regions will decrease in magnitude during physiological aging; however, other studies report that changes in EEG recordings of mild cognitive impairments and AD experience a change in pattern. Alpha and beta rhythms usually decrease, while there is a general increase in delta and theta oscillations. These findings have been described as “a slowing of the EEG”, which is correlated with a decreased state of arousal and cognitive processing. It has been shown that the slowing of an EEG could be linked with the atrophy reported in AD patients of cholinergic neurons in the basal forebrain which innervates both the neocortex and the hippocampus [35]. On account of EEG signals being non-stationary, complex, and nonlinear signals, higher complexity measures in EEGs are believed to reflect the integration of information among isolated groups of neurons, which are performing different processing tasks; reduced complexity would reflect a lower degree of information exchanged. Therefore, the analysis of complexity would provide a measure of the amount of information which is being integrated within a neural system. Thus, if there is a reduction in EEG complexity within AD patients it can be interpreted as an alteration in the information being exchanged, which has been reported in multiple studies [35]. The synchronization between neuronal populations is pertinent to the interaction between neural networks, and EEG synchrony is referencing the adjustment of different neural oscillations. Two different signals would become coupled (synchronized) when they both begin oscillating at the same frequency, different measures are used to quantify the synchronization of neurons and neural activity. One of these measurements is spectral coherence, which corresponds to the spectral covariance of the activity between the two electrode locations [35]. Studies have shown that AD patients had reduced resting state EEG coherence when compared to healthy controls. Delbeuck et al. [36] postulate that the pathophysiological reasons underlying the synchrony loss in patients with AD is due to the degenerative processes that are caused by the disease. The neurofibrillary tangles and amyloid-beta plaques would physically interrupt the flow of electricity between long cortico-cortical tracts, which would inherently lead to a neocortical disconnection between neurons. It has also been postulated that the perturbation in EEG synchrony could originate from a loss in cortical neurons, in combination with reduced cholinergic activity in the cortices of patients with AD [35].

EEG is a non-invasive and low-cost technique which would constitute a good alternative compared to current biomarker techniques being used. Taking into consideration the complexity of AD pathophysiology, the current knowledge surrounding EEG-derived biomarkers suggest that it could reach accuracy, sensitivity, and specificity for the diagnosis of AD, as well as estimation of the severity and progression of the disease [35].

## 5. Current Research in Alzheimer’s Disease: Microglia, LEDs, and Electromagnetic Fields 

A recent development in AD pathogenesis research is the identification of the role of Apolipoprotein E (APOE) in AD and age-related cognitive impairment [37,38,39]. APOE is a crucial protein in lipid metabolism, specifically facilitating the transport of cholesterol and other lipids within the body. In the central nervous system (CNS), glial cells, particularly microglia, have been identified as expressing APOE [37]. The APOE gene contains instructions for APOE synthesis, leading to different isoforms, with the most common being apoE2, apoE3, and apoE4. These APOE isoforms are of particular interest in AD and other dementias due to their critical roles in disease development and potential protective features [40]. Notably, APOE3 (Christchurch) and APOE4 (Jacksonville) play essential roles, with opposing effects. APOE4 is implicated in disease progression, while recent evidence suggests that APOE3 may have protective effects [41]. In the brain, APOE is involved in synaptic formation, tissue repair, and neurite outgrowth, all crucial factors for optimal cognitive functioning. Understanding the distinct contributions of APOE isoforms is vital in identifying their impact on neurodegenerative disorders, particularly AD and related dementias. In a recent study, Chen and colleagues [42] conducted experiments demonstrating that an APOE3 mutation in a mouse model significantly reduces tau pathology. The modulation of microglial response seems to play a crucial role in this observed reduction in tau pathology. Specifically, the expression of APOE appears to influence how microglia react to Aβ plaques, ultimately inhibiting the seeding and propagation of Aβ-induced tau.

Beyond microglia and gene mutations, recent studies have also focused on entraining gamma waves in the brain through light flickering [31,32,33,34,35,42,43]. In experiments, mice with AD pathologies were exposed to flickering light, and their behavior and brain function were assessed afterward. Specifically, flickering light stimulation (FLS) at 40 Hz significantly reduces AD pathologies in mice, leading to reduced amyloid and tau protein levels, prevention of cerebral atrophy, and improved behavioral testing performance [30]. Pushing forward into human testing, recent human studies show promising results with less ventricular dilation, hippocampal atrophy, increased functional connectivity, and improved cognitive and daily activity measures after three months of daily 40 Hz stimulation [30]. The central tenet of these papers is that applying a 40 Hz frequency can lead to a noticeable increase in gamma activity in the brain, potentially influencing cognitive functioning.

The notion of observing individual cell types or proteins in AD research may be counterproductive. A more effective approach for understanding and treating AD could involve studying the aging brain as a system. This perspective emphasizes a holistic methodology, focusing on the entire system rather than isolated examinations of specific cell types or proteins. Research published within the last few years has shown novel therapeutics being used against AD pathologies, yielding successful results. The application of both EMFs and light-emitting diodes (LED), oscillating at 40 Hz, has been proven to significantly decrease amyloid-beta plaques and AD pathologies in mice, respectively [44,45]. The frequency of 40 Hz was chosen as it is the same frequency as a gamma wave, a fast oscillating brain wave that is highly correlated with cognitive functioning and has been shown to significantly decrease in patients with AD and MCIs [46]. Utilizing EMFs and LEDs as potential therapeutic agents could be extremely beneficial, as they are non-invasive and come without adverse effects.

EMFs constitute dynamic forces carrying both magnetic and electric components, existing wherever an electric current flows [47]. Different from static fields like those generated by a fridge magnet, the dynamic EMFs described in this review have varying characteristics over time, a crucial distinction. The impact and effect of EMFs on biological systems is a complex subject. Depending on the frequency, duration, and intensity of the EMF presented, a variety of effects could be observed [47]. Previous research has demonstrated EMFs’ diverse effects on biological systems, from inhibiting cancer cell growth in a dish to influencing nociception in rats [48,49]. Additionally, Lefaucheur and colleagues [50] have leveraged EMFs, specifically repetitive transcranial magnetic stimulation (rTMS), as a non-invasive technique to influence brain activity. Additional experiments by Hoogendam and colleagues [51] have explored lasting effects dependent on factors like frequency, intensity, duration, and pulse structure. The nature of the applied EMF is pivotal, comparable to different pharmacological agents and their respective dosages. The literature consistently indicates that EMFs have the potential to impact biological systems across various levels of discourse. However, determining the optimal combination of duration, pattern, and intensity remains an unresolved aspect in current research. 

Dragicevic and colleagues [52] efficiently showed that long-term exposure to high-frequency EMF treatment in Alzheimer’s transgenic (Tg) mice not only prevents cognitive impairment, but also reverses it and improves memory functioning in normal mice. The EMF treatment was able to disaggregate amyloid-beta peptide (Aβ) oligomers, which are the form of Aβ that causes mitochondrial dysfunction in AD [52]. The study showed that EMF treatment provides cognitive benefits to both transgenic and non-transgenic mice, via the primary mechanism of brain mitochondrial enhancement [52]. Additionally, the impact of adulthood EMF exposure was studied by Arendash et al. [53] which showed that daily EMF treatment given to very old mice (21–27 months) over a 2-month period not only reverses the very advanced Aβ aggregation and deposition in their brain, but also caused an increase in general memory function. This study shows that EMF exposure can be effective later in the progression of the disease and not just at the beginning. Another demonstration of EMF treatment providing general cognitive benefit to incredibly old Alzheimer’s transgenic and normal mice [53].

In humans with cognitive decline, with both MCI and AD, a novel therapeutic tool Emisymmetric bilateral stimulation (EBS) emits noise-like stimuli using EMF to trigger self-arrangements in the cortex, thereby improving cognitive faculties [54]. After only 5 weeks of standardized EBS therapy, significant improvements were observed in all neurocognitive assessments (Mini-metal state examination, assessment of episodic memory, executive functions, and behavioral disorders). Another promising, effective, safe, and non-invasive tool to be used to combat cognitive impairment [54]. Patients with AD can experience impairments in visual memory and visuoconstructive functions. Sandyk [55] reported that the exogenous application extremely low intensity and frequency EMF improved both visual memory and visuoperceptive functions in patients with Parkinson’s disease. There is a subgroup of patients with Parkinson’s who have coexisting pathological and clinical features of AD, so the extremely weak EMF were applied to patients with AD [55]. Patients who received the EMF had a dramatic improvement in visual memory and enhancement of visuoconstructive performance, which was associated (clinically) with improvement in other cognitive functions, such as short-term memory, calculations, spatial orientation, judgement, and reasoning, as well as energy levels, social interactions, and overall mood. These results of the rapid improvement in cognitive functions resulting from the EMF suggest that the mental deficits of AD are caused by a functional rather than a structural disruption of neuronal communication on the central nervous system [55].

Finally, recent studies have demonstrated that EMF application has the capacity to selectively target microglia, eliciting neuroprotective effects against AD [56,57]. In a specific investigation, EMF application was observed to enhance the expression of heat shock proteins, leading to a subsequent positive interaction between microglia and astrocytes. This crosstalk initiated favorable effects such as neurorestoration [58]. A summary of EMF and LED research can be seen in Table 1. 

## 6. The Future and Concluding Remarks

In this review, various pieces of research and intervention have been examined, each offering some relief from the symptoms of AD. While several approaches show promise, none currently provide a complete reversal of the diseases. Despite this, our comprehension and treatment options are advancing, with the identification of APOE mutations potentially representing a significant breakthrough in understanding disease onset and prevention. This is particularly highlighted by the research of Chen and colleagues [42], who effectively replicated the original human case report by Arboleda-Velasquez et al. [38] using mice in the laboratory. Research focusing on the modulation of APOE and microglia needs to persist and undergo further exploration. This includes the identification of additional mechanisms capable of influencing microglia to induce neuroprotective effects, recognizing that future treatment strategies for AD and cognitive decline may not rely on a singular stimulus. LED and EMF research, also shows promise but may benefit from combination with other features, potentially involving microglia through APOE modulation. In recent years, several research groups have showcased the interaction between microglia and EMF application [56,57,58]. The manipulation of microglia through EMF application offers the potential for synergistic effects when combined with additional methodologies, maximizing the positive impacts of each mechanism.

Bioengineering and biomaterials also present a significant avenue for addressing the complexities of AD. Utilizing methodologies such as organoids, spheroids, and other cutting-edge techniques, researchers are gaining deeper insights into disease progression, diagnostics, and treatment strategies. Through these innovative approaches, advanced diagnostic tools capable of detecting AD in its earliest stages are being developed, facilitating timely interventions. Integration of bioengineering and biomaterials into AD research heralds a new era of investigation, poised to make substantial advancements in addressing this challenging neurodegenerative disorder.

Given that our behaviors are intricately linked to complex biochemical changes and cerebral dynamics, it is crucial to recognize the multifaceted nature of AD intervention. Singular approaches focusing on isolated mechanisms may prove counterintuitive. While studying individual proteins or cell types is indispensable, true understanding of disease progression and pathology necessitates a broader perspective. This does not diminish the importance of focused research; instead, it emphasizes the significance of understanding the “big picture.” In the future, holistic approaches that include biomaterials could play a crucial role in neurorestoration and combating cell pathologies associated with the progression of AD. These biomaterials might be manipulated using various methods such as EMFs, LFS, nanoparticles, or a combination of proteins to stimulate cell function. It is essential to subject these biomaterials to testing within holistic systems like organoids and spheroids. Doing so enhances diagnostic capabilities, deepens our understanding of disease progression, and expands treatment possibilities. An interdisciplinary approach to understanding how behavioral changes arise from dynamic cerebral alterations and protein modifications is essential. Neuroscience provides the foundation for this initiative. Embracing diverse methodologies will be central to effectively diagnosing, understanding, and treating AD.

## Figures and Tables

**Table 1:** Evidence linking electromagnetic fields and light-emitting diodes as therapeutic agents for Alzheimer’s disease.

Reference	Study	Outcome
Jensen et al., 2021 [59]	Study investigated the effects of transcranial bipolar pulsed electromagnetic fields on motor performance in patients with idiopathic Parkinson’s disease.	Long-term treatment with transcranial bipolar pulsed electromagnetic fields significantly increased movement and speed.
Arendash et al., 2022 [60]	Patients with AD received daily transcranial electromagnetic treatment (TEMT).	Results showed that TEMT administration was completely safe and had no deleterious side effects. Also showed that TEMT can stop the cognitive decline of AD without safety issues.
Cichon et al., 2020 [61]	Studied the effect of an extremely low-frequency electromagnetic field (ELF-EMF) on the molecular mechanism of apoptosis to study the rehabilitation of post-stroke patients.	Exposure to ELF-EMF significantly increased the expression of pro-apoptotic genes in post-stroke patients which promotes the activation of signaling pathways involved in brain plasticity processes.
Faraji et al., 2021 [62]	Fifty male adult rats were exposed to extremely low-frequency electromagnetic fields (ELF-EMFs) to study the impact on memory, anxiety, antioxidant activity, beta-amyloid deposition, and microglia population.	Exposure to ELF-EMF had an anxiogenic effect on rats, promoted memory, and induced oxidative stress. No amyloid-beta depositions were detected in the hippocampus of groups who received ELF-EMF.
Cao et al., 2022 [63]	Patients with (AD) received transcranial electromagnetic stimulation (TEMT) in order to study the effects on blood or cerebrospinal fluid (CSF) levels of 12 cytokines to further study the link between AD and the immune system.	Patients who received daily TEMT had balanced cytokines, which was associated with the reversal of their cognitive impairments.
Kazemi et al., 2022 [64]	Studied the effect of extremely low-frequency electromagnetic fields (ELF-EMFs) on the structure and function of the brain in male rhesus monkeys. Studying visual learning (VL), visual memory (VM), and visual working memory (VWM).	After exposure to ELF-EMFs, the abilities of VL, VM, and VWM significantly improved.. It was found that ELF-EMFs irradiations can improve cognitive abilities in monkeys.
Pooam et al., 2021 [65]	Photobiomodulation and pulsed electromagnetic fields were used as noninvasive therapies in HEK293 cells to study the effect on inflammation and ROS signaling pathways related to COVID-19.	Cells that were exposed to a moderate-intensity infrared light significantly lowered the inflammatory response. Cells that were exposed to either pulsed electromagnetic fields or low-level static magnetic fields also achieved similar anti-inflammatory effects.
Kim et al., 2019 [66]	Studied the effect of pulsed electromagnetic field therapy (PEMF) on blood pressure and nitric oxide in subjects with mild to moderate metabolic syndrome.	The PEMF group showed an increase in nitric oxide after therapy when compared to controls.
Gomez et al., 2021 [67]	The protective effect of pulsed electromagnetic field s(PEMF) was studied in nerve growth factor-differentiated pheochromocytoma PC12 cells that had been injured with 48 hours of hypoxia.	Exposure to the PEMF reduced cell death by 13%, PEMF also triggered p39 kinase phosphorylation and stimulated the cytoprotective chaperone molecule HSP70. PEMF also increased CREB phosphorylation and restored BDNF basal levels.
Khajei et al., 2021 [68]	Kindled rats were exposed to electromagnetic fields (EMF) to study the effects of EMF on learning and memory, as well as hippocampal synaptic plasticity.	Applying EMF to the kindling and EMF group restored learning and memory, and decreased escape latency and path length in the Morris water maze. Electrophysiological results showed that EMF returned the ability of synaptic potentiation to the hippocampus and improved memory formation.
Gao et al., 2021 [69]	Rat models of middle cerebral artery occlusion/reperfusion were treated with extremely low-frequency electromagnetic fields (ELF-EMF) to study its effect on cerebral ischemia.	Rats treated with ELF-EMF required shorter swimming distances and latencies in Morris water maze. findings in the study suggest that ELF-EMF can enhance the hippocampal neurogenesis in rats with cerebral ischemia.
Nazari et al., 2022 [70]	Study to determine the effect of 40 Hz white light LED on the structure–function of mitochondrial ATP-sensitive potassium channel and brain mitochondrial respiratory chain activity, production of reactive oxygen species (ROS), and mitochondrial membrane potential in AD.	Noninvasive exposure to 40 Hz white light LED increased activities of complexes I and IV and decreased ROS production and mitochondrial membrane potential up to 70%.
Cho et al., 2018 [71]	Investigated the effects of photobiomodulation using LEDs on amyloid plaques, gliosis, and neuronal loss to prevent and/or recover cognitive impairments in 5XFAD mice that were used as an AD model.	Results showed photobiomodulation treatment at earlier stages reduced amyloid accumulation, neuronal loss, and microgliosis as well as alleviating cognitive dysfunction in 5XFAD mice.
Wang et al., 2020 [72]	Explored the effect of LED therapy on myocardial ischemia–reperfusion (IR) injury on thirty rats, measuring via electrocardiogram. Brain and heart tissue were extracted for myocardial injury.	Rats with IR injury that received LED therapy had a significant decrease in ischemia size and infarct size when compared to the IR injury group that did not receive LED. It was found that LED therapy might reduce neuroinflammation in the paraventricular nucleus and decrease myocardial injury.
Mansano et al., 2021 [73]	Effects of light-emitting diodes (LEDs) on mesenchymal stem cells (MSCs) were studied via 10 articles.	LEDs have shown to induce greater cell viability, proliferation, differentiation, and secretion of growth factors of MSCs.
Choi et al., 2023 [74]	Evaluated the safety and efficacy of LED therapy in management of pain and stiffness with refractory hand tenosynovitis, 12 patients received LED therapy twice a week for four weeks.	After LED therapy patients showed clinically significant improvements, compared to their baseline, in visual analog scale scores at weeks 2, 4, and 8. Proving LED therapy as a viable alternative to pharmacological treatments.
Siqueira, 2024 [75]	Studied the potential for LED devices as treatment for retinal degenerative disease. Rat models with rhodopsin mutations were administered daily LED treatment for five days during photoreceptor development.	Study showed that LED treatment increased mitochondrial cytochrome oxidase activity, upregulated the production of antioxidant protective enzymes and cofactors, enhanced the production of neurotrophic factors, and even prevented apoptotic cell death in the photoreceptors.
Stepanov et al., 2022 [76]	Oligomeric beta-amyloid was prepared and used to treat microglial cells and light irradiation of cells was performed using diode lasers.	Light induces metabolic shift during co-cultivation of neurons with microglia; light prevents the death of neurons.

## Data Availability

No new data were created or analyzed in this study. Data sharing is not applicable to this article.
